# Composition and Antioxidant Status of Vegan Milk—Pilot Study

**DOI:** 10.3390/antiox14050505

**Published:** 2025-04-23

**Authors:** Agnieszka Chrustek, Agnieszka Dombrowska-Pali, Dorota Olszewska-Słonina

**Affiliations:** 1 Department of Pathobiochemistry and Clinical Chemistry, Faculty of Pharmacy, Collegium Medicum in Bydgoszcz, Nicolaus Copernicus University, M. Curie-Skłodowskiej 9, 85-094 Bydgoszcz, Poland; a.chrustek@cm.umk.pl (A.C.); dorolsze@cm.umk.pl (D.O.-S.); 2 Department of Perinatology, Gynecology and Gynecologic Oncology, Faculty of Health Sciences, Collegium Medicum in Bydgoszcz, Nicolaus Copernicus University, Łukasiewicza 1, 85-821 Bydgoszcz, Poland

**Keywords:** human milk, vegans, antioxidants, nutrition

## Abstract

Background: More and more women are following a vegan and vegetarian diet. For some, the use of a vegan diet during lactation is controversial. Purpose: The aim of the study was to comparatively analyze the concentration of selected hormones, micro- and macronutrients, vitamins, and the basic composition and antioxidant status of the milk of vegan women, compared to the milk of omnivorous women. Methods: The study included 17 breastfeeding vegan women and 27 omnivorous women. The basic composition of human milk was analyzed using the MIRIS HMATM analyzer (Uppsala, Sweden) The levels of hormones and vitamins were determined by the enzyme-linked immunosorbent method. In order to determine the antioxidant activity and micro- and macroelements, spectrophotometric methods were used. Results: The vegan group was characterized by a lower average age, lower BMI, and lower WHR index compared to the control group. The milk of vegan women showed significantly higher cortisol concentrations and lower iron, vitamin B6, and antioxidant status than the milk of omnivorous women. Conclusions: A vegan diet helps maintain a healthy body weight and is more popular among younger women, under 30 years of age. Higher levels of milk cortisol in vegan women may indicate a high level of anxiety and stress experienced by breastfeeding women, which may have negative consequences not only for breastfeeding mothers but also for the development of their children. Lack of appropriate supplementation in women who do not consume meat and animal products may cause a deficiency of micro- and macroelements in breast milk.

## 1. Introduction

Proper nutrition during pregnancy and lactation is crucial for the health of both the mother and the child. A balanced diet rich in essential nutrients supports the development of the fetus and then ensures adequate milk production. Human milk is usually sufficient to cover the caloric requirements of the child and thus support its growth and development. It is widely believed that the mother’s diet affects the composition of human milk, especially the fatty acid profile, which is well documented [1,2,3]. However, the remaining variability of nutrients and macro- and microelements in human milk attributable to diet remains largely unknown [4]. It should be noted that restrictive dietary restrictions of lactating women may affect the nutrient content of human milk as well as reduce the volume of milk produced [5]. In the context of optimal nutrient intake, the use of a vegan diet by breastfeeding women is controversial. The vegan diet is the most restrictive form of vegetarianism. Vegans eliminate all animal products from their diet. In recent years, it has been observed that plant-based diets are becoming increasingly popular in Europe [6]. It is estimated that the prevalence of vegans in Europe is from 1 to 10% [7]. In turn, in 2024, according to a report conducted by CEOWORLD Consumer Insights magazine, Poland ranked 19th in the world in terms of the population of vegetarians and vegans, 8.4% and 1.8%, respectively [8]. The more restrictive the vegan diet, the greater the risk of dietary deficiencies. Hence, doubts have arisen as to the adequacy of nutrient intake by vegan women and the impact of this diet on the composition of breast milk [9].

According to the American Academy of Nutrition and Dietetics, the composition of vegan milk is similar to that of non-dieting mothers, except for the concentration of fatty acids. They recommend this diet, properly balanced, with well-chosen supplementation [10]. The Swiss Federal Food Commission and the German Nutrition Society have expressed an opposing position, claiming that a vegan diet cannot be recommended for pregnant and breastfeeding women nor for infants, children, and the elderly [11,12]. According to the guidelines of the European Milk Bank Association (EMBA), breastfeeding vegan women can be recruited as donors to the Human Milk Bank, provided that they regularly supplement vitamin B12 [13]. In turn, national guidelines for individual countries vary; for example, in Switzerland, Germany, and Australia, vegan mothers cannot donate their milk to other children [14]. In Poland, a vegan diet is a permanent exclusion criterion for donating human milk [15]. Given the scientific evidence for the varied intake of some nutrients during a vegan diet, which may translate into differences in the composition of human milk, the aim of this study was to conduct a comparative analysis between the milk of vegan women and the milk of women consuming all animal products.

Human milk consists, among others, of carbohydrates, fats, proteins, bioactive factors (immunoglobulins, hormones, growth factors), vitamins, micro- and macronutrients, as well as the microbiome [16,17,18,19,20,21,22].

Carbohydrates are utilized in energy processes but also for the proper maturation of the nervous system and the maintenance of a healthy bacterial flora in the gastrointestinal tract of infants [16]. Human milk oligosaccharides (HMOs) exhibit anti-adhesive, anti-infective, and probiotic properties [17]. Meanwhile, proteins in human milk not only provide nutrition but also serve bioactive functions. They act as carriers of other nutrients (e.g., lactoferrin, alpha-lactalbumin, β-casein), regulate intestinal development (e.g., growth factors, lactoferrin), and nutrient absorption (e.g., bile salt-stimulated lipase, amylase), and demonstrate immunological and antimicrobial activity (e.g., lactoferrin, secretory IgA, cytokines, lysozyme) [18,23]. Fats constitute the primary energy source (approximately 50% of calories, equivalent to 25 g per day up to 6 months of age) and are an important source of essential nutrients such as polyunsaturated fatty acids (PUFAs), fat-soluble vitamins, complex lipids, and bioactive compounds [18]. The lipids in human milk positively influence child development. Long-chain polyunsaturated fatty acids (LCPUFAs) stimulate the functional development of the retina and brain in infants, participate in inflammatory and immune processes, which affects the optimal maturation of the immune system, and may additionally influence adipocyte differentiation and contribute to reducing the risk of obesity [19].

Bioactive factors in human milk include immunoglobulins, hormones, growth factors, glycans, nucleotides, blood cells, cytokines, stem cells, lactoferrin, antioxidant compounds such as vitamins A, E, C, β-carotene, and glutathione, as well as antioxidant enzymes (e.g., glutathione peroxidase, catalase, superoxide dismutase) [20,21,24,25,26].

Human milk contains numerous hormones, including thyroid hormones (thyroxine, triiodothyronine), thyrotropin (TSH), thyrotropin-releasing hormone (TRH), parathormone, cortisol, melatonin, progesterone, estrogens, prolactin, insulin, adipose tissue hormones (adiponectin, resistin, leptin, ghrelin), and growth hormone [16,20,25,26]. Cortisol (a stress hormone, glucocorticoid) is produced by the adrenal cortex, influences metabolism, and possesses anti-inflammatory properties. It is a crucial component of human milk due to its role in regulating the digestive system and influencing the neurological development of the child [20].

Adipose tissue hormones have a positive effect on early satiety control in infants and the regulation of energy balance during childhood and adulthood, protecting against later obesity. These hormones are likely secreted and synthesized by the mammary gland and pass from the serum into human milk. Adipose tissue hormones present in human milk include leptin, adiponectin, resistin, ghrelin, obestatin, irisin, apelin, and visfatin [25,26]. Adiponectin has anti-inflammatory and anti-atherogenic properties and regulates metabolism [25], while leptin improves insulin sensitivity, regulates energy balance, and appetite [26].

Human milk is rich in all vitamins, micro- and macroelements necessary for the proper development of the child. Macronutrients include Na, Cl, K, Mg, Ca, phosphates, and sulfates, while microelements include Fe, Cu, Mn, Zn, Co, Mo, Se, Cr, F, and I [24,27,28,29]. Vitamin B6 supports the body’s immune resistance, participates in gluconeogenesis and antibody formation, and aids the child’s nervous system [27]. Vitamin D is involved in bone formation and plays a crucial role in calcium–potassium balance. Its deficiency causes rickets in children [30].

Due to the limited scientific literature on the impact of veganism on the composition and antioxidant status of human milk, the aim of our study was to assess the basic composition, hormones (adiponectin, leptin, cortisol), vitamins (vitamin D, vitamin B6), micro- and macroelements (iron, phosphorus, magnesium, calcium), and the antioxidant status of vegan mothers’ milk.

## 2. Materials and Methods

The research was approved by the Bioethics Committee of the Nicolaus Copernicus University in Toruń at the Ludwik Rydygier Collegium Medicum in Bydgoszcz (consent no. KB437/2018).

All women participating in the study read information about it, completed the questionnaire, and expressed their conscious opinion, written consent to participate in it. The questionnaire included questions about the age of women, residence, professional activity, weight and height of a breastfeeding woman, duration of pregnancy, fertility (primiparous/multiparous), type of delivery, diet, and taking medications and supplements.

### 2.1. Characteristics of the Vegan and Control Groups

The study group initially included 23 vegan women and 27 women who consumed all animal products. In the group of vegan women recruited for the study, only 5 women consciously supplemented vitamin B12. The remaining 17 women only used supplementation due to breastfeeding when recommended by medical personnel. Finally, the study included 17 women who had excluded all animal products such as meat, milk, honey, dairy products, and eggs from their diet for at least 3 years and did not supplement vitamin B12.

**Figure 1:**
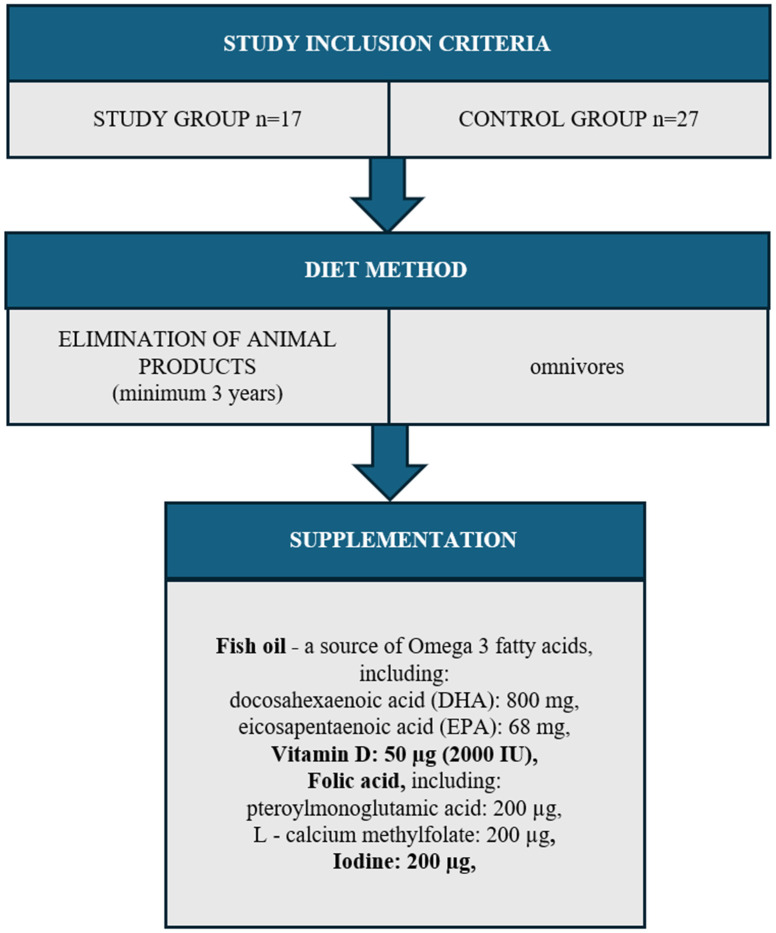
Study group inclusion criteria.

Both the study group (n = 17) and the control group (n = 27) excluded women with diagnosed diseases: thyroid, circulatory system, digestive system, respiratory system, cancer, and allergies. The study also did not include women addicted to nicotine or women taking any medications. The entire study group came from one region of the Kujawsko-pomorskie Voivodeship (Poland). The women resided in urban areas and held higher education degrees. They were primiparous, having given birth via vaginal delivery, and were in the 3rd to 6th month of lactation. The mothers exclusively breastfed their infants without supplementation with formula or solid foods (Figure 1).

The material for the study was human milk from breastfeeding women. The study participants volunteered to participate in the research project via social media in response to the information about the study. Human milk came from a daily collection (40 mL). Each woman expressed milk using a breast pump according to the daily milk collection protocol four times a day, from four time intervals: 06:00–12:00, 12:00–18:00, 18:00–24:00, and 24:00–6:00. The desired amount of expressed milk from each time interval was 10 mL, with 5 mL before putting the baby to the breast and 5 mL after the end of feeding. Each portion of milk was poured into one collective bottle. The material was provided by breastfeeding women within 24 h of collection, then aliquoted into 2 mL Eppendorf tubes (MedLab, Raszyn, Poland) and frozen at −20 °C and then at −80 °C. The material was stored in these conditions until the parameters described below were determined, but no longer than 3 months.

### 2.2. Determination of the Basic Composition of Breast Milk

The basic composition of breast milk includes the content of fats [mg/100 mL], total protein [mg/100 mL], crude protein [mg/100 mL], carbohydrates [mg/100 mL], total solids [mg/100 mL], and energy value [kcal/100 mL]. The determination was performed using the MIRIS HMAT analyzer (MIRIS AB, (Uppsala, Sweden)) in accordance with the manufacturer’s procedure. Miris HMA™ software (2022) processes the measurement data via internal calibration. Detection limit: for fat content—0.06 g/100 mL, crude protein—0.06 g/100 mL, total protein—0.05 g/100 mL, and for carbohydrates—0.04 g/100 mL.

Breast milk samples were heated at 40 °C in a thermostatic bath (BIONOVO, Legnica, Poland) before analysis and then homogenized with MIRIS Sonicator (Białystok, Poland) [1.5 s/mL]. Each sample was analyzed in three replicates.

### 2.3. Determination of Leptin Concentration

In order to determine leptin, a ready-made, commercial enzyme-linked immunosorbent assay, Human Leptin ELISA (BioVender, Karásek, Czech Republic) was used in accordance with the manufacturer’s procedure. This method consisted of 60 min of incubation of milk samples and standards (100 μL each) on a 96-well plate coated with polyclonal antibodies against human leptin. The next step was the application of 100 μL of polyclonal antibody conjugated to horseradish peroxidase and incubation for 60 min. The resulting conjugate reacted with 100 μL of TMB, then the reaction was stopped by the addition of an acidic solution (100 μL of sulfuric acid VI). The absorbance was measured using the MultiSkan Go (Vantaa, Finland) reader at a wavelength of 450 nm relative to the reference wave (630 nm). Detection limit: 0.2 ng/mL. Each sample was analyzed in three replicates.

### 2.4. Determination of Adiponectin Concentration

In order to determine adiponectin in human milk, a commercial enzyme-linked immunosorbent assay (Human Adiponectin/Acrp30, DuoSet ELISA, R&D Systems, Minneapolis, MN, USA) was used.

Prior to analysis, a 96-well plate was coated with antibodies and incubated overnight at room temperature, then rinsed, and a reagent containing 1% BSA (bovine serum albumin) in PBS was added and incubated for 1 h. After this time, 100 μL of human milk and standard samples were applied and incubated for 2 h, then the plate was rinsed and a detection antibody (100 μL) was added, incubated for another 2 h, washed, and 100 μL Streptavidin-HRP was added and incubated for 20 min. In the next step, the plate was rinsed, 50 μL of “Substrate Solution” (containing TMB) was added and incubated for 20 min at room temperature to protect it from light, finally “Stop Solution” (containing acid) was added to stop the reaction, the plate was gently shaken and the absorbance was measured at a wavelength of 450 nm using the MultiSkan Go reader. Detection limit: 10 pg/mL. Each sample was analyzed in three replicates.

### 2.5. Determination of Cortisol Concentration

A commercial enzyme-linked immunosorbent assay, Cortisol Elisa (DiaMetra, Via Pozzuolo, Italy), was used to determine cortisol in human milk according to the manufacturer’s procedure. Human milk samples and standards (20 μL each) were applied to a 96-well plate coated with antibodies, followed by the addition of 200 μL of antigen-conjugated horseradish peroxidase solution, and incubated at 37 °C for 60 min. Subsequently, 100 μL of TMB was added to obtain a colored solution, incubated for 15 min at room temperature in the dark, and the reaction was stopped by the application of sulfuric acid VI (100 μL). Absorbance was read at a wavelength of 450 relative to the reference wave (620–630 nm) using the MultiSkan Go reader. Detection limit: 0.12 ng/mL. Each sample was analyzed in three replicates.

### 2.6. TAS Marking

A ready-to-use, commercially sourced enzyme-linked immunosorbent assay (TAS, München, Germany) was used to determine the total antioxidant capacity in accordance with the manufacturer’s procedure. A 10 μL sample of human milk, a calibrator, and a light were applied to the 96-well plate. A 100 μL reagent containing a peroxide solution was added and prepared and then incubated for 10 min at 37 °C. Reagent with an enzyme was added to some wells, and a reagent without an enzyme was added to the rest. Then, 50 μL of “Stop Solution” (containing sulfuric acid) was added to stop the reaction. The absorbance at a wavelength of 450 nm was read.

The total antioxidant capacity was calculated using the formula:

$$TAS=392-(392-calibrator\;concentration)\times \left(\frac{change\;in\;absorbance\;of\;the\;sample}{change\;in\;absorbance\;of\;the\;calibrator}\right)\;[\mu mol/L]$$

Reference Range:

<250 μmol/L—low antioxidant capacity

250–300 μmol/L—medium antioxidant capacity

>300 μmol/L—high antioxidant capacity

Each sample was analyzed in three replicates.

### 2.7. Determination of the Antioxidant Activity of Human Milk Using the DPPH• (2,2-Diphenyl-1-picrylhydrazyl) Radical

The method of Atanassov et al. was used in the study. (2011) with minor modifications [31].

Prior to the experiment, a 100 μM solution of DPPH• (2,2-diphenyl-1-picrylhydrazyl, Sigma Aldrich, Darmstadt, Germany) was prepared by dissolving 4 mg of the standard substance in 100 mL of methanol (POCH, Gliwice, Poland).

Briefly, 1 mL of prepared DPPH solution was applied to Eppendorf tubes (Warsaw, Poland), 250 μL of human milk was added, vortexed, and then incubated for 60 min in the dark at room temperature. Before reading, the samples were centrifuged for 2 min at 1500× *g*, at room temperature. The absorbance was measured at a wavelength of 517 nm against the reference sample, which was methanol. The control sample was a 100 μM methanol DPPH solution, which was measured at the beginning and end of the experiment. The solutions for obtaining the curve were prepared in a similar way, replacing 250 μL of human milk with Trolox solutions (Morąg, Poland).

Each sample was analyzed in three replicates.

**Preparation of the calibration curve**

Five solutions of Trolox (TE, (+−)-hydroxy-2,5,7,8-tetramethylchromatane-2-carboxylic acid, Sigma Aldrich, Darmstadt, Germany) at concentrations of 10–80 mg/1000 mL were prepared to determine the ability to deactivate free radicals in human milk. The calibration curve shows the dependence of the absorbance value of Trolox on its concentration. The results were expressed in terms of Trolox equivalent (μmol TE/1000 mL, mg TE/100 mL). In addition, the percentage ability of human milk to reduce the DPPH radical was calculated:

$$\%inhibition=(\frac{A-Ab}{A})\times 100$$

A—control absorbance;

Ab—average absorbance value of the tested human milk;

Detection limit: 0.1 μg TE/mL.

### 2.8. Determination of the Ability of Human Milk to Reduce Fe (III) Ions

In the experiment, the FRAP method (iron ion reduction ability) according to Benzie and Strain (1999) was used with modifications [32].

Labeling in human milk rolls

Before the experiment, the following were prepared:

(a) 300 mM acetate buffer, pH 3.6;
(b) 10 mM TPTZ (2,4,6-Tri(2-pyridyl)-s-triazine, Sigma-Aldrich, Darmstadt, Germany) in 40 mM HCl;
(c) 20 mM FeCl_3_ × 6H_2_O. (Chempur, Piekary Śląskie, Poland).

The first step was to prepare the FRAP reagent by combining solutions a, b, and c in a ratio of 10:1:1. For the purpose of determination, a blank sample was prepared, which was a FRAP reagent, and 100 μL of human milk samples and calibration curve standards. The next step was to apply 100 μL of sample and 3 mL of FRAP reagent, vortexing, followed by immediate reading of sample absorbance at 0 min at 593 nm and incubation at 37 °C in a water bath for 4 min. The last stage was vortexing and subsequent measurement of absorbance at the same wavelength.

Each sample was analyzed in three replicates.

**Preparation of the calibration curve**

Six solutions of ascorbic acid (Chempur, Piekary Śląskie, Poland) with concentrations of 100–1000 μM were prepared in order to determine the ability to reduce Fe(III) ions in human milk. The standard curve shows the dependence of the absorbance value of ascorbic acid on its concentration. FRAP concentrations are expressed as the equivalent of ascorbic acid (μM).

The FRAP value is calculated from the formula:

$$FRAP=\frac{change\;in\;sample\;absorbance\;from\;0\;min\;to\;4\;min}{change\;in\;absorbance\;of\;the\;standard\;from\;0\;min\;to\;4\;min}\times standard\;concentration\times 2\;[\mu M]$$

Ascorbic acid has a constant stoichiometric coefficient of 2.0 in the FRAP test.

Detection limit: 2 μM.

### 2.9. Determination of the Content of the Total Sum of Polyphenols

**Determination of human milk samples**

The method of Vazquez et al. was used for the determination (2015) with modifications [33]. The milk samples were gradually thawed in the refrigerator and vortexed. The first stage of the assay was the deproteinization of the samples by mixing 1 mL of a sample of human milk with 2 mL of 50% methanol, 100 μL of Carrez I and Carrez II reagent, and 1 mL of acetonitrile The samples were then vortexed and topped up with 50% methanol to a volume of 5 mL. The samples were incubated at room temperature for 25 min, and finally centrifuged at 4500× *g* at 4 °C for 15 min.

The total sum of polyphenols was determined in the aqueous layer of the sample by the FC (Folin–Ciocâlteu, Chempur, Poland) method. Briefly,100 μL of methanol extract was mixed with 1 mL of previously prepared FC reagent (FC:H20; 1:1, *v*/*v*) and with 3 mL of 20% Na_2_CO_3_. After vortexing, the mixture was incubated for 30 min in the dark at room temperature. The absorbance was then measured at a wavelength of 765 nm using a UV/VIS spectrophotometer (Biosense, Wrocław, Poland). The solutions necessary to obtain the calibration curve were prepared by replacing 100 μL of methanolic extract with gallic acid solutions (Merck, Darmstadt, Germany) of known concentration. The reference sample was 100 μL of methanol.

Each sample was analyzed in three replicates.

**Preparation of the calibration curve**

Six aqueous standard solutions of gallic acid (GAE) were prepared with concentrations: 0.0–0.10 mg/mL. The total polyphenol content was calculated on the basis of a calibration curve describing the dependence of absorbance values on the concentration of gallic acid. Results are presented as the equivalent of gallic acid in 1000 mL of human milk (mg GAE/L).

Detection limit: 5 mg GAE/L.

### 2.10. Determination of Paraoxonase 1 (PON1) Concentration

In order to determine the concentration of paraoxonase 1, a ready-made, commercial enzyme-linked immunosorbent assay (Human Paraoxonase 1 ELISA, BioVender, Karásek, Czech Republic) was used. Standards and samples of human milk (100 μL each) were incubated on a 96-well plate coated with polyclonal antibodies, then after 60 min of incubation (37 °C) and washing, 100 μL of biotin-labeled antibody was applied and incubated for another 60 min at 37 °C. The next stage was rinsing and an application of 100 μL of Streptavidin-HRP and another 30 min incubation (37 °C), then the plate was rinsed for the last time, and 100 μL of TMB and 10 min incubation were added. The last step was to stop the reaction by adding 100 μL of sulfuric acid (VI) each. The absorbances were read at a wavelength of 450 nm relative to the reference wave (620–630 nm). Detection limit: 0.1 ng/mL. Each sample was analyzed in three replicates.

### 2.11. Determination of Iron Concentration

A set of reagents from BioMaxima (Lublin, Poland) was used for the determination of iron (Table 1).

Making the marking:

A ready-made standard from BioMaxima (Lublin, Poland) (200 μg/dL) was used.

The concentration of iron in the human milk samples was calculated using the formula:

$$Iron\;concentration=\frac{A2(PB)-A1(PB)}{A2(PW)-A1(PW)}\times standard\;concentration\;[\mu g/dL]$$

A1(PB)—absorbance of 1 test sample;

A2(PB)—absorbance of 2 test sample;

A1(PW)—absorbance of standard 1;

A2(PW)—absorbance 2 of the standard;

Each sample was analyzed in three replicates.

### 2.12. Determination of Magnesium, Calcium, and Phosphorus Concentration

In order to determine magnesium, calcium, and phosphorus, the BioMaxima kit (Lublin, Poland) was used. Each sample was analyzed in three replicates (Table 2).

Making the marking:


$$Magnesium\;concentration=[mg/dL]\frac{A\;(PB)}{A(PW)}\times standard\;concentration$$

A(PB)—absorbance of the test sample;

A(PW)—standard absorbance.

### 2.13. Determination of Vitamin D Concentration

To determine the concentration of vitamin 25(OH)D3 (HVD3) in human milk, a commercial enzyme-linked immunosorbent assay from Cloud-Clone Corp (Katy, TX, USA) was used. The test was performed in accordance with the rules of the procedure given by the test manufacturer. Briefly, 50 μL of standards and human milk samples were pipetted onto a 96-well plate, and then 50 μL of reagent A was added to each well and incubated for 1 h at 37 °C. Subsequently, the plate was rinsed 3 times, and 100 μL of reagent B was added at 37 °C. The penultimate stages were rinsing the plate and adding 90 μL of “Substrate Solution” (incubation 15 min, 37 °C). Finally, 50 μL of “Stop Solution” was added to each well, and the absorbance at a wavelength of 450 nm was read. Detection limit: 4.83 pg/mL. Each sample was analyzed in three replicates.

### 2.14. Determination of Vitamin B6

In order to determine the concentration of vitamin B6 in human milk, an enzyme-linked immunosorbent assay from Cloud-Clone Corp. (USA) was used. The principle of the method and the performance of the test are identical to the above-described test for the determination of vitamin D.

Detection limit: 1.00 ng/mL. Each sample was analyzed in three replicates.

### 2.15. Statistical Analysis

In order to carry out the statistical analysis, the Statistica 13.1 software package from StatSoft^®^ (Kraków, Poland) was used.

The normality of the schedule was verified by the Shapiro–Wilk test. No normality of the distribution of the analyzed quantitative variables was found.

A nonparametric Mann–Whitney U test was used to assess statistical significance in two groups of independent variables. The variability of the parameters is presented in the form of a median, minimum, and maximum (Min–Max).

In order to analyze many factors per variable in the studied groups, multiple linear regression was used. The variability of the parameters is presented in the form of a regression coefficient, error of the standard, regression coefficient, and the level of statistical significance *p* < 0.05.

To assess the correlation between the parameters studied, the Spearman correlation test was used. The results at the level of *p* < 0.05 were considered statistically significant.

The correlations that occur are represented by a correlation matrix.

## 3. Results

The group of vegan women had a lower mean age, lower BMI, and lower WHR compared to the control group (Table 3).

### 3.1. Basic Composition of Human Milk

No significant differences were observed in the parameters of the basic composition of human milk between the group of vegans and the control group (Table 4).

### 3.2. Hormones in Human Milk

No significant differences were observed in the concentration of adipose tissue hormones (adiponectin, leptin) in human milk between the vegan group and the control group (Table 3). A median increase in cortisol concentration of 61.10% (*p* = 0.022) in the milk of vegan women was shown compared to the control group (Table 5).

### 3.3. Antioxidant Status of Human Milk

The antioxidant status of vegan women shows significant differences compared to the milk of women who do not follow a diet. In the group of vegans, the median total antioxidant status of human milk was lower by 32.82% (*p* < 0.001) and the ability of human milk to reduce iron ions by 27.50% (*p* = 0.049), as well as the concentration of paraoxonase 1 by 29.03% (*p* = 0.021) and polyphenols by 11.18% (*p* = 0.039), compared to the control group (Table 6).

In the group of vegans, high positive correlations were found between BMI/DPPH (r = 0.556, *p* = 0.020) and WHR/polyphenols (r = 0.659; *p* = 0.007), and high negative correlations between age/FRAP (r = −0.553; *p* = 0.021) and cortisol/TAS (r = −0.557; *p* = 0.020) (Tables S1–S3).

### 3.4. Content of Vitamins, Micro-, and Macroelements in Human Milk Samples

The content of iron and vitamin B6 in human milk differs significantly in the group of vegans (*p* = 0.037; *p* = 0.024) compared to the control group. The median concentration of iron in human milk is lower by 41.88% in the study group compared to the control group (*p* = 0.037), while vitamin B6 is lower by 3.49%. The remaining micro- and macroelements examined did not show any significant statistical differences (Table 7).

In the group of vegans, a high negative correlation was observed between magnesium and leptin (r = −0.565; *p* = 0.018) and calcium-polyphenols (r = −0.514; *p* = 0.035). In addition, high positive correlations were found between vitamin B6 and adiponectin (r = 0.747; *p* = 0.001) and vitamin D and calcium (r = 0.641; *p* = 0.007), (Tables S1–S3).

### 3.5. Analysis of the Influence of Different Predictors on the Tested Parameters

No significant statistical difference was found when examining multiple linear regressions between the examined parameters (DPPH, FRAP, cortisol, fat, total protein, carbohydrates, total solids, caloric value) and predictors (HBD, BMI, WHR, age, stage of lactation, type of delivery, place of residence) in the study groups.

## 4. Discussion

Breast milk is sufficient for the development of a child in most cases due to its rich composition and adaptation to the needs of the child. Permanent dietary restrictions during lactation may result in the exhaustion of the breastfeeding woman’s body and negatively affect both the volume of breast milk and the content of certain nutrients in breast milk. Of great importance for the development of a child in the first months are proteins, especially amino acids, polyunsaturated fatty acids, vitamin D, B vitamins, iron, and iodine [34]. A vegan diet is often accused of not providing the right amount of nutrients [12].

Our studies did not show any significant differences in parameters such as BMI, HBD, but a positive correlation was observed between HBD and WHR (r = 0.601; *p* < 0.001). These results indicate that younger women are adopting veganism and that diet and HBD can affect the figure of a breastfeeding woman.

There is little scientific research on the effect of a vegan diet on the composition of human milk. According to literature data, a vegan and vegetarian diet may affect the composition of breast milk, mainly the fatty acid composition and vitamin concentration [5,9,35].

Milk from vegetarian women contains reduced levels of saturated long-chain fatty acids and higher levels of polyunsaturated fatty acids [5]. Sanders et al. (1978) [35] showed significantly lower amounts of C16:0, C16:1, and C18:0, and higher C18:2 and C18:3 in the milk of vegan women compared to women not following a diet. A higher percentage of ALA (2.09%) was shown in milk from vegan women compared to milk from vegetarians (1.55%) and from women not following a diet (1.19%). There was no statistically significant difference in the concentration of DHA in the study groups (*p* = 0.543) [21]. Patel and Lovelady (1998) observed a higher amount of short-chain fatty acids in the milk of vegan women compared to the milk of vegetarians and women who did not follow a diet, similarly for linoleic acid and α-linolenic acid [36]. The amount of long-chain fatty acids in human milk was higher in the milk of omnivorous women than in the milk of vegetarians and vegans. Compared to other dietary groups, the share of DHA in the milk of vegan women was lower, and in addition, a lower LA/ALA ratio and a higher omega-6/omega-3 ratio were observed in the milk of vegan women than in the other groups [35]. The results of our own studies did not show significant differences in the basic composition of human milk between the group of vegans and the group of omnivorous women.

Further studies show a lower concentration of taurine in the milk of vegan women (35 mg/dL) compared to the milk of omnivorous women (53 mg/dL) [37,38]. Due to the various guidelines regarding the vegan diet during breastfeeding, mainly caused by reports of insufficient demand for vitamins, micro-, and macroelements during the above diet, it was decided to extend our own research by analyzing selected compounds in human milk. Our own studies showed that the concentration of iron in the milk of vegan women is 41.88% lower compared to the control group, while the content of phosphorus, magnesium, and calcium does not differ significantly. There is no reference to the results in the scientific literature, but there are a few studies comparing the iron status in the blood plasma of vegans to people who do not follow a diet. Scientists have shown that the iron concentration between the two groups does not differ significantly, but it was noted that vegans have lower plasma ferritin concentrations compared to the control group [39]. Low levels of ferritin, an iron-binding protein, may be related to insufficient iron storage, which in turn leads to lower concentrations in breast milk.

Concerned about the content of vitamins, micro-, and macroelements, researchers have studied the effect of diet on this topic in a rather narrow scope. It was shown that the concentration of vitamin B12 in milk from vegetarian women was significantly lower (*p* = 0.006) [36]. Similar results were obtained by Specker et al. (1990) [40]. Pawlak et al. (2018) [41] analyzed the concentration of a given vitamin in human milk from vegans, vegetarians, and women who did not follow a diet, and no significant differences were observed. The results of our own studies showed that the milk of vegans contains a lower concentration of vitamin B6 compared to the control group (*p* = 0.024), while no significant differences were observed with regard to vitamin D (*p* = 0.398). There is a lack of comparative literature regarding the content of vitamin B6 in human milk in the groups discussed, but we can refer to the content of this substance in blood plasma. Atamasirikul et al. (2008) [42] confirm the effect of a vegan diet on the content of vitamin B6 in the human body, showing a lower median concentration of the substance in the blood plasma of vegans (37.40 nmol/L) compared to people not following a diet (47.40 nmol/L). Schüpbach et al. (2017) [43] had the opposite opinion, showing no effect of diet on the concentration of vitamin B6 in the body of vegans (27 nmol/L), but they showed lower values in vegetarians (16 nmol/L) compared to the control group (22 nmol/L).

These data draw particular attention to the precise preparation of the meal in vegans and possible deficiencies in supplementation.

To date, the scientific literature does not contain studies comparing the content of hormones in human milk, as well as the antioxidant status of human food between the groups described above. The results of our own studies showed an increase in the median concentration of cortisol in milk from vegan women (9.36 ng/mL) compared to the control group (5.81 ng/mL). Its increased concentration may indicate increased stress factors affecting vegans, but it should also be remembered that cortisol in appropriate concentrations stimulates the digestive system in children. In this study, a decrease in TAS in daytime samples and FRAP, polyphenol concentration, and PON1 in nighttime samples of milk from vegan women was also observed. These results indicate lower protection against free radicals and, consequently, in the future against free radical diseases, such as cancer, cardiovascular diseases, diabetes, and neurodegenerative diseases. These results are surprising considering the fact that vegans consume foods rich in antioxidants. On the other hand, analyzing our own results showing deficiencies in the content of vitamin B6, which inhibits oxidative stress [44,45], suggests a significant effect of the content of a given vitamin on antioxidant status. 

## 5. Conclusions

A plant-based diet is becoming increasingly popular. People who do not consume meat and animal products have a lower body mass index (BMI) and are slimmer than people who eat meat.

Our research indicates that the elimination of meat and animal products alone does not have a significant effect on the nutritional composition of breast milk. Vegan mothers are able to produce nutritionally valuable milk for their infants. This is particularly significant for vegan women during lactation, as their dietary choices should not serve as a permanent exclusion criterion for their participation as donors in human milk banks—a practice that currently occurs, among other places, in Poland. However, the results of this study draw attention to the need to introduce recommendations for supplementation in breastfeeding women who follow a vegan diet. The milk of women who follow a vegan diet, in the absence of appropriately selected supplementation, has lower values of micro- and macroelements and a lower antioxidant status, which may affect the growth and development of the child. Higher levels of milk cortisol were also observed in the breast milk of vegan women, indicating that these women experienced more long-term stress than omnivores. Therefore, special attention should be paid to the mood of a postpartum woman who follows a vegan diet, as the higher levels of milk cortisol in our study probably indicate that the elimination diet itself is a risk factor for long-term stress, which may consequently affect mental health.

## Figures and Tables

**Table 1:** Determination of iron concentration in human milk.

	Reagent Test	Human Milk Samples	Pattern
R1 reagent	750 μL	750 μL	750 μL
Standard/Sample	-	75 μL	75 μL
Distilled water	75 μL	-	-
The samples were thoroughly mixed, incubated for 5 min at 37 °C, centrifuged for several seconds at the highest speed, and then the absorbance A1 was read against the reagent test at a wavelength of 590 nm.			
R2 Reagent	75 μL	75 μL	75 μL
The samples were mixed, incubated at 37 °C for 5 min, after which time the samples were centrifuged for several minutes at the highest speed, and then the A2 absorbance was read against the reagent test at a wavelength of 590 nm.			

**Table 2:** Determination of magnesium, calcium, and phosphorus concentrations in human milk.

	Reagent Test	Human Milk Samples	Pattern
R1 reagent	750 μL	750 μL	750 μL
Standard/Sample	-	75 μL	75 μL
Distilled water	75 μL	-	-
The samples were thoroughly mixed, incubated for 5 min at 37 °C, centrifuged for several seconds at the highest speed, and then the absorbance A1 was read against the reagent test at a wavelength of 590 nm.			
R2 Reagent	75 μL	75 μL	75 μL
The samples were mixed, incubated at 37 °C for 5 min, after which time the samples were centrifuged for several minutes at the highest speed, and then the A2 absorbance was read against the reagent test at a wavelength of 590 nm.			

**Table 3:** Characteristics of the study groups.

	Vegans n = 17	Control Group n = 27	*p*
Age (years) $\bar{x}\pm SD$	29.23 ± 4.29	30.74 ± 2.68	**0.049**
BMI (kg/m^2^) (Me; Min–Max)	21.26; 17.31–27.14	23.05; 17.93–29.30	0.066
HBD (week) (Me, Min–Max)	40.00; 35.00–42.00	40.00; 35.00–41.00	0.425
WHR (Me, Min–Max)	0.78; 0.71–0.85	0.81; 0.72–1.06	**0.039**

n—size; SD—standard deviation; BMI—body mass index [kg/m$\bar{x}$^2^]; WHR—ratio of waist circumference to hip circumference; HBD—week of pregnancy; Me—median; Min–Max—minimum and maximum values; *p*—level of statistical significance (Mann–Whitney U test).

**Table 4:** Comparison of the basic composition of human milk in vegan and non-dieting women.

Variable	Study Group: Vegans n = 17 Me (Min–Max)	Control Group: Women Not Following Diets n = 27 Me (Min–Max)	*p*
Fat [g/100 mL]	3.40 (1.30–6.90)	3.30 (0.80–7.300)	0.847
Total protein [g/100 mL]	1.20 (0.40–1.60)	1.20 (0.70–1.50)	0.615
Carbohydrates [g/100 mL]	7.90 (6.60–8.30)	7.80 (6.20–8.30)	0.734
Total solids [g/100 mL]	12.60 (10.10–16.30)	12.20 (10–16.00)	0.847
Energy value [g/100 mL]	68 (47–101)	65 (44–100)	0.426
Crude protein [g/100 mL]	1.00 (0.30–1.30)	0.90 (0.50–1.20)	0.419

Me—median; Min–Max—minimum and maximum values; *p*—level of statistical significance (Mann–Whitney U test); n—number.

**Table 5:** Hormone content in the milk of vegans and women who do not follow a diet.

Variable	Study Group: Vegans n = 17 Me (Min–Max)	Control Group: Women Not Following Diets n = 27 Me (Min–Max)	*p*
Adiponectin [pg/mL]	3233 (540–5230)	2900 (335–7891)	0.735
Leptin [ng/mL]	1.52 (1.04–10.46)	1.65 (1.10–9.06)	0.628
Cortisol [ng/mL]	9.36 (3.97–48.02)	5.81 (1.48–16.29)	**0.022**

Me—median; Min–Max—minimum and maximum values; *p*—level of statistical significance (Mann–Whitney U test); n—number.

**Table 6:** Antioxidant status of human milk in vegan and non-dieting women.

Variable	Study Group: Vegans n = 17 Me (Min–Max)	Control Group: Women Not Following Diets n = 27 Me (Min–Max)	*p*
TAS [μM]	200.13 (156.60–272.99)	298.03 (170.99–403.96)	**<0.001**
DPPH [% Inhibition]	47.35 (4.37–76.30)	53.24 (24.95–74.91)	0.419
DPPH [μM TE/L]	208.50 (68.60–302.76)	227.67 (135.59–298.21)	0.419
FRAP [μM]	482.97 (65.10–887.21)	666.16 (155.94–2272.51)	**0.049**
PON1 [ng/mL]	1.10 (0.46–2.12)	1.55 (0.46–4.46)	**0.021**
Polyphenols [mg GAE/L]	10.33 (7.40–23.98)	11.63 (7.08–102.064)	**0.039**

TAS—total antioxidant status; FRAP—iron ion reduction ability; DPPH—2,2-diphenyl-1-picrylhydrazyl radical reduction method; TE—trolox; GAE—gallic acid; PON1—paraoxonase 1; Me—median; Min–Max—minimum and maximum values; *p*—level of statistical significance (Mann–Whitney U test), n—abundance.

**Table 7:** The content of selected vitamins, micro-, and macronutrients in the milk of vegans and women who do not follow a diet.

Variable	Study Group: Vegans n = 17 Me (Min–Max)	Control Group: Women Not Following Diets n = 27 Me (Min–Max)	*p*
Iron [μg/dL]	8.52 (1.38–21.63)	14.66 (1.98–65.01)	***p* = 0.037**
Phosphorus [mg/dL]	6.26 (2.25–12.64)	5.16 (2.57–7.45)	*p* = 0.231
Magnesium [mg/dL]	4.02 (2.74–8.73)	4.02 (2.51–5.94)	*p* = 0.774
Calcium [mg/dL]	14.06 (8.29–32.82)	13.90 (6.79–21.88)	*p* = 0.703
Vitamin D [pg/mL]	138.60 (62.05–804.87)	162.10 (44.38–354.40)	*p* = 0.398
Vitamin B6 [ng/mL]	273.20 (1.05–296.80)	283.10 (155.50–350.89)	***p* = 0.024**

*p*—level of statistical significance (Mann–Whitney U test).

## Data Availability

Data will be made available upon request. Data are stored at the Department of Pathobiochemistry and Clinical Chemistry, Collegium Medicum in Bydgoszcz (Poland). Person responsible for providing data: Agnieszka Chrustek (mail: a.chrustek@cm.umk.pl).

## Supplementary Materials

The following supporting information can be downloaded at: https://www.mdpi.com/article/10.3390/antiox14050505/s1.

## Abbreviations

ALA	alpha-lipoic acid
BMI	Body Mass Index
DHA	docosahexaenoic acid
DPPH	2,2-diphenyl-1-picrylhydrazyl radical reduction method
FRAP	iron ion reduction ability
GAE	gallic acid
HBD	hebdomas graviditatis
LA	lipoic acid
Me	median
Min–Max	minimum and maximum values
n	abundance
*p*	level of statistical significance
PON1	paraoxonase 1
TAS	total antioxidant status
TE	trolox
WHR	waist-to-hip ratio
