# Supplementary material for: Composition and Antioxidant Status of Vegan Milk—Pilot Study
Source: Antioxidants (Basel). 2025 Apr 23;14(5):505. doi: 10.3390/antiox14050505 (PMC12108482; doi:10.3390/antiox14050505)
Supplement: Supplementary file 1 [file antioxidants-14-00505-s001.zip › antioxidants-3543302-supplementary.pdf]

Table SI Spearman rank order correlation matrices significant with **$p<0.05$** in the control group (n=27).

Variable	Age	BMI	Adi po Nect in	Leptin	Cortisol	TAS	DPPH	FRAP	PON1	Fat	Total protein	carbohydrates	Dry Mass Wodany	Energy value	HBD
Age	1.000	0.019	0.048	-0.155	0.058	-0.248	-0.139	-0.285	-0.023	-0.047	-0.278	0.026	-0.067	-0.135	-0.188
BMI	0.019	1.000	-0.280	-0.001	0.163	-0.199	0.145	0.083	-0.014	0.139	-0.293	0.211	0.162	0.197	-0.099
Adiponectin	0.048	-0.280	1.000	0.093	-0.045	0.017	-0.066	-0.127	-0.322	-0.147	0.053	-0.393	-0.234	-0.283	0.346
Leptin	-0.155	-0.001	0.093	1.000	0.460	-0.239	-0.008	-0.205	-0.195	0.022	0.172	0.263	0.068	0.135	0.179
Cortisol	0.058	0.163	-0.045	0.460	1.000	-0.474	-0.169	-0.474	-0.136	0.190	0.065	0.149	0.200	0.295	0.131
TAS	-0.248	-0.199	0.017	-0.239	-0.474	1.000	0.364	0.095	0.213	-0.002	0.128	-0.212	0.021	-0.044	-0.020
DPPH	-0.139	0.145	-0.066	-0.008	-0.169	0.364	1.000	-0.052	0.191	0.189	0.231	-0.118	0.202	0.128	-0.417
FRAP	-0.285	0.083	-0.127	-0.205	-0.474	0.095	-0.052	1.000	0.181	-0.199	0.037	0.217	-0.128	-0.100	-0.260
PON1	-0.023	-0.014	-0.322	-0.195	-0.136	0.213	0.191	0.181	1.000	0.207	-0.253	-0.091	0.179	0.222	-0.479
Fat	-0.047	0.139	-0.147	0.022	0.190	-0.002	0.189	-0.199	0.207	1.000	0.191	0.160	0.961	0.874	-0.271
Total protein	-0.278	-0.293	0.053	0.172	0.065	0.128	0.231	0.037	-0.253	0.191	1.000	0.257	0.289	0.219	0.157
Carbohydrates	0.026	0.211	-0.393	0.263	0.149	-0.212	-0.118	0.217	-0.091	0.160	0.257	1.000	0.346	0.329	-0.215
Total solids	-0.067	0.162	-0.234	0.068	0.200	0.021	0.202	-0.128	0.179	0.961	0.289	0.346	1.000	0.913	-0.288
Energy value	-0.135	0.197	-0.283	0.135	0.295	-0.044	0.128	-0.100	0.222	0.874	0.219	0.329	0.913	1.000	-0.222
HBD	-0.188	-0.099	0.346	0.179	0.131	-0.020	-0.417	-0.260	-0.479	-0.27	0.157	-0.215	-0.288	-0.222	1.000
WHR	0.173	0.397	-0.299	0.370	0.068	0.018	0.291	0.013	0.055	-0.231	-0.290	-0.009	-0.147	-0.072	-0.086
Vitamin D	-0.078	0.396	-0.086	0.149	0.054	-0.006	0.079	-0.475	-0.475	-0.309	0.147	0.213	-0.190	-0.225	0.454
Vitamin B6	-0.110	0.231	-0.126	-0.054	0.219	-0.139	-0.168	0.312	0.212	-0.120	-0.156	0.104	-0.109	0.020	-0.203
Magnesium	-0.392	0.040	0.170	0.506	0.156	0.142	0.400	0.096	-0.153	0.043	0.224	0.015	0.059	0.028	-0.081
Phosphorus	-0.097	-0.007	0.169	-0.069	-0.032	0.191	0.055	-0.148	-0.269	0.014	-0.138	-0.160	0.007	0.022	0.098
Calcium	0.093	0.434	0.027	0.162	0.068	0.104	0.164	-0.231	-0.047	-0.182	-0.184	-0.013	-0.185	-0.280	0.132

BMI – body mass index; TSH – thyrotropin [mU/l]; TAS – total antioxidant status [µM]; FRAP – iron ion reduction capacity [µM]; DPPH – 2,2-diphenyl-1-picrylhydrazyl radical reduction method [% inhibition]; PON1 – paraoxonase 1 [ng/ml]; HBD – week of pregnancy; WHR – ratio of waist circumference to hip circumference; adiponectin, melatonin – hormone concentration in pg/ml; leptin cortisol – hormone concentration in ng/ml; fat, total protein, carbohydrates, total solids, energy value – content in human milk in g/100ml; phosphorus, magnesium, calcium – concentration in mg/dl; vitamin D – concentration in pg/ml; vitamin B6 – concentration in ng/ml

Table SII Correlation matrices of the Spearman rank order relevant with **$p<0.05$** in the group of vegans (n=17).

Variable	Age	BMI	Adiponectin	Leptin	Cortisol	TAS	DPPH	FRAP	PON1	Fat	Total protein	Carbohydrates	Energy value	HBD	WHR
Age	1.000	0.021	0.201	-0.080	0.205	-0.145	-0.437	-0.553	-0.248	-0.049	-0.209	-0.067	-0.046	0.089	-0.170
BMI	0.021	1.000	-0.039	0.333	-0.075	0.084	0.556	-0.219	0.123	0.159	0.180	-0.124	0.066	0.333	0.146
Adiponectin	0.201	-0.039	1.000	-0.180	-0.183	0.166	0.004	0.194	-0.201	-0.125	-0.195	-0.320	-0.127	0.008	0.078

Leptin	-0.080	0.333	-0.180	1.000	0.007	0.074	0.177	-0.161	0.195	-0.081	-0.347	-0.211	-0.185	0.309	0.203
Cortisol	0.205	-0.075	-0.183	0.007	1.000	-0.557	-0.286	-0.272	-0.324	0.309	0.044	-0.101	0.316	0.188	-0.135
TAS	-0.145	0.084	0.1667	0.074	-0.557	1.000	0.210	0.058	0.463	-0.072	0.297	-0.219	-0.111	-0.032	-0.030
DPPH	-0.437	0.556	0.004	0.177	-0.286	0.210	1.000	0.172	0.096	0.527	0.523	0.202	0.568	-0.051	0.200
FRAP	-0.553	-0.219	0.194	-0.161	-0.272	0.058	0.172	1.000	0.179	-0.048	-0.046	0.132	-0.074	-0.353	0.300
PON1	-0.248	0.123	-0.201	0.195	-0.324	0.463	0.096	0.179	1.000	-0.034	-0.003	-0.185	-0.133	-0.143	-0.112
Fat	-0.049	0.159	-0.125	-0.081	0.309	-0.072	0.527	-0.048	-0.034	1.000	0.414	-0.066	0.893	-0.259	0.071
Total protein	-0.209	0.180	-0.195	-0.347	0.044	0.297	0.523	-0.046	-0.009	0.414	1.000	0.364	0.621	-0.110	-0.278
Carbohydrates	-0.067	-0.124	-0.320	-0.211	-0.101	-0.219	0.202	0.132	-0.185	-0.066	0.364	1.000	0.230	-0.184	-0.012
Total solids	-0.018	0.008	-0.148	-0.265	0.251	-0.107	0.555	-0.058	-0.142	0.783	0.709	0.398	0.966	-0.320	-0.130
Energy value	-0.046	0.066	-0.127	-0.185	0.316	-0.111	0.568	-0.074	-0.133	0.893	0.621	0.230	1.000	-0.346	-0.121
HBD	0.089	0.333	0.008	0.309	0.188	-0.032	-0.051	-0.353	-0.143	-0.259	-0.110	-0.184	-0.346	1.000	0.601
WHR	-0.170	0.146	0.078	0.203	-0.135	-0.030	0.200	0.300	-0.112	0.071	-0.278	-0.012	-0.121	0.601	1.000

BMI – body mass index; TSH – thyrotropin [mU/l]; TAS – total antioxidant status [µM]; FRAP – iron ion reduction capacity [µM]; DPPH – 2,2-diphenyl-1-picrylhydrazyl radical reduction method [% inhibition]; PON1 – paraoxonase 1 [ng/ml]; HBD – week of pregnancy; WHR – ratio of waist circumference to hip circumference; adiponectin, melatonin – hormone concentration in pg/ml; leptin cortisol – hormone concentration in ng/ml; fat, total protein, carbohydrates, total solidssolids, energy value – content in human milk in g/100ml

Table SIII Correlation matrices of the Spearman rank order relevant to **$p<0.05$** in the group of vegans (n=17).

Variable	Age	BMI	Adiponectin	Leptin	Cortisol	TAS	DPPH	FRAP	PON1	Fat	Total protein	Carbohydrates	Total solids	Energy value
Iron	-0.010	0.421	-0.312	0.606	0.042	-0.080	0.136	-0.355	0.137	0.127	-0.261	-0.058	-0.086	0.056
Magnesium	0.036	-0.031	0.105	-0.565	-0.127	0.164	-0.100	0.410	0.056	0.149	0.013	0.055	0.012	0.049
Phosphorus	-0.257	-0.090	-0.210	-0.300	0.284	-0.329	-0.034	0.052	-0.332	0.323	0.143	0.098	0.194	0.228
Calcium	-0.111	0.279	-0.058	0.072	-0.068	-0.118	0.036	-0.285	0.110	-0.378	0.042	0.320	-0.154	-0.214
Vitamin d	0.036	0.326	-0120	0.402	-0.300	0.257	0.235	-0.376	0.238	-0.168	0.006	-0.050	-0.163	-0.127
Vitamin B6	0.024	-0.083	0.747	0.159	-0.282	0.199	-0.122	0.093	0.353	-0.346	-0.500	-0.262	-0.355	-0.325

BMI – body mass index; TSH – thyrotropin [mU/l]; TAS – total antioxidant status [µM]; FRAP – iron ion reduction capacity [µM]; DPPH – 2,2-diphenyl-1-picrylhydrazyl radical reduction method [% inhibition]; PON1 – paraoxonase 1 [ng/ml]; HBD – week of pregnancy; WHR – ratio of waist circumference to hip circumference; adiponectin, melatonin – hormone concentration in pg/ml; leptin cortisol – hormone concentration in ng/ml; fat, total protein, carbohydrates, total solids, energy value – content in human milk in g/100ml; iron – concentration in µg/dl; magnesium, phosphorus, calcium – concentration in mg/dl; vitamin D – concentration in pg/ml; vitamin B6 – concentration in ng/ml
